# People with Type 2 Diabetes Mellitus (T2DM) Self-Reported Views on Their Own Condition Management Reveal a High Level of Insight into the Challenges Faced

**DOI:** 10.1177/19322968211009261

**Published:** 2021-05-17

**Authors:** Mike Stedman, Rustam Rea, Mark Livingston, Katie McLoughlin, Louise Wong, Stephen Brown, Katherine Grady, Roger Gadsby, Angela Paisley, Adrian Heald

**Affiliations:** 1 Res Consortium, Andover, Hampshire; 2 Oxford Centre for Diabetes, Endocrinology and Metabolism and NIHR Oxford Biomedical Research Centre, Oxford University Hospitals NHS FT, Oxford; 3 Department of Clinical Biochemistry, Black Country Pathology Services, Walsall Manor Hospital, Walsall; 4 School of Medicine and Clinical Practice, Faculty of Science & Engineering, The University of Wolverhampton; 5 Salford Royal Hospital, Salford, UK; 6 Warwick Medical School, University of Warwick, Coventry, UK; 7 The School of Medicine and Manchester Academic Health Sciences Centre, University of Manchester, UK

**Keywords:** blood glucose, monitoring, HbA1c, type 2 diabetes, patient experience, survey

Finger prick blood glucose (BG) monitoring remains a mainstay of management in people with type 2 diabetes mellitus (T2DM) who take sulphonylurea (SU) drugs/insulin, although it has not been found to improve outcomes in people not taking these agents.^1^

We recently examined patient experience of BG monitoring in type 1 diabetes mellitus (T1DM).^2,3^ There has not been any similar recent assessment in people with T2DM in the UK. This study aimed to address this matter.

A digital questionnaire containing 49 questions was sent by email to patients on the Research for the Future (RftF) consent for approach database.^4^

Of those individuals approached to complete the online questionnaire, 25.5% (186) responded. 84% were treated with insulin plus other hypoglycemic agents. When asked about glycemic control, 51% of patients self-reported their last HbA1c as ≤64 mmol/mol/8.0%. 7% reported last HbA1c to be >86 mmol/mol/10.0%. 75% reported having an HbA1c check in the previous 6 months.

We next asked about how people with T2DM felt about the relationship between BG levels/insulin dosing, 30% stated that they keep BG level high sometimes to avoid hypoglycemia. Furthermore, 52% were concerned they might be over-/under-dosing their insulin.

In relation to shorter-term consequences of high BG levels, for HbA1c ≥65 mmol/mol/8.1%, a significant proportion (70%) were concerned/really concerned versus not concerned/undecided about the consequences of running a high HbA1c. In contrast, of those not knowing their HbA1c only 33% were concerned/really concerned about the consequences of high HbA1c (Figure 1).

**Figure 1.**
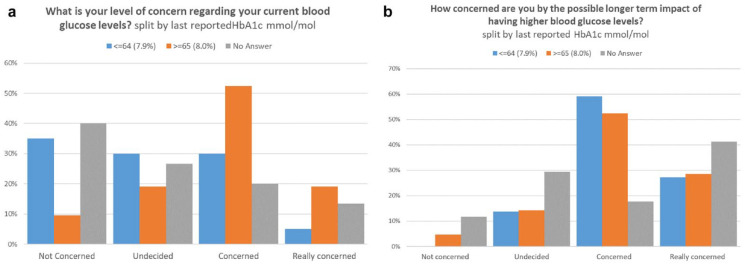
(a) Level of concern about current blood glucose levels split by last reported glycated hemoglobin A1c (HbA1c). (b) Level of concern about future blood glucose levels split by last reported HbA1c.

For longer term consequences in relation to HbA1c for both HbA1c ≤64 mmol/mol/8.0% (86%) and ≥65 mmol/mol/8.1% (81%), a high proportion of people were concerned (59%) about their BG readings, as did those not knowing their HbA1c (Figure 1).

When questioned about adequacy of information about BG monitoring, only 25% said they had sufficient information, with 43% believing that accuracy and precision of their BG meter was being independently checked. Only 9% remembered discussing BG meter accuracy when their latest meter was provided. Only 7% were aware of the International Standardisation Organisation (ISO) standards for BG meters.^5^

Encouragingly, 75% reported having had an HbA1c check in the last 6 months although this survey was carried out before the Coronavirus pandemic which has resulted in many HbA1c tests not being performed at the appropriate time.^6^

Thus, in this group of people with T2DM who are engaged with their diabetes management, long term concerns (as in T1DM)^3^) were highly prevalent in contrast to views of current BG levels where there was overall less concern, particularly in those not recalling their HbA1c. We found significant concern about over- or under-dosing of insulin. Only one-quarter of patients responded that they had sufficient information about BG monitoring. This indicates a large gap in patient education.

The group surveyed comprised long-term engaged people with T2DM. Even within this group there was significant variation in (a) awareness of shorter-term risks, (b) confidence in their ability to implement appropriate insulin dosage, (c) awareness of the limitations of BG monitoring technology. These are clearly areas where additional education/support would provide significant benefit.

## Supplemental Material

sj-tif-1-dst-10.1177_19322968211009261 – Supplemental material for People with Type 2 Diabetes Mellitus (T2DM) Self-Reported Views on Their Own Condition Management Reveal a High Level of Insight into the Challenges Faced Click here for additional data file. Supplemental material, sj-tif-1-dst-10.1177_19322968211009261 for People with Type 2 Diabetes Mellitus (T2DM) Self-Reported Views on Their Own Condition Management Reveal a High Level of Insight into the Challenges Faced by Mike Stedman, Rustam Rea, Mark Livingston, Katie McLoughlin, Louise Wong, Stephen Brown, Katherine Grady, Roger Gadsby, Angela Paisley and Adrian Heald in Journal of Diabetes Science and Technology

sj-tif-2-dst-10.1177_19322968211009261 – Supplemental material for People with Type 2 Diabetes Mellitus (T2DM) Self-Reported Views on Their Own Condition Management Reveal a High Level of Insight into the Challenges Faced Click here for additional data file. Supplemental material, sj-tif-2-dst-10.1177_19322968211009261 for People with Type 2 Diabetes Mellitus (T2DM) Self-Reported Views on Their Own Condition Management Reveal a High Level of Insight into the Challenges Faced by Mike Stedman, Rustam Rea, Mark Livingston, Katie McLoughlin, Louise Wong, Stephen Brown, Katherine Grady, Roger Gadsby, Angela Paisley and Adrian Heald in Journal of Diabetes Science and Technology
