# Supplementary figures and images for: People with Type 2 Diabetes Mellitus (T2DM) Self-Reported Views on Their Own Condition Management Reveal a High Level of Insight into the Challenges Faced
Source: J Diabetes Sci Technol. 2021 May 17;15(4):972–3. doi: 10.1177/19322968211009261 (PMC8252150; doi:10.1177/19322968211009261)

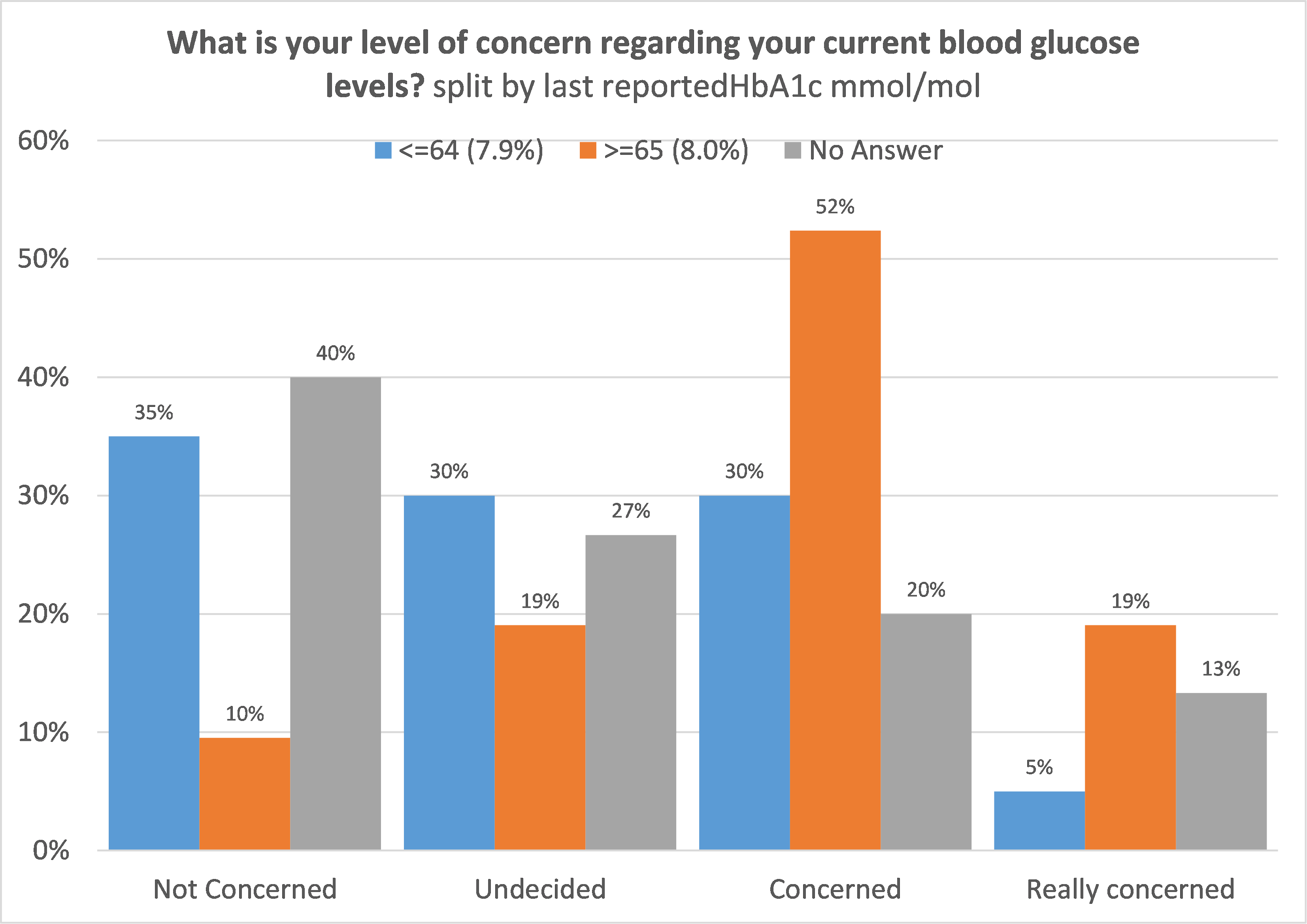

Supplement: sj-tif-1-dst-10.1177_19322968211009261 – Supplemental material for People with Type 2 Diabetes Mellitus (T2DM) Self-Reported Views on Their Own Condition Management Reveal a High Level of Insight into the Challenges Faced [file sj-tif-1-dst-10.1177_19322968211009261.tif]

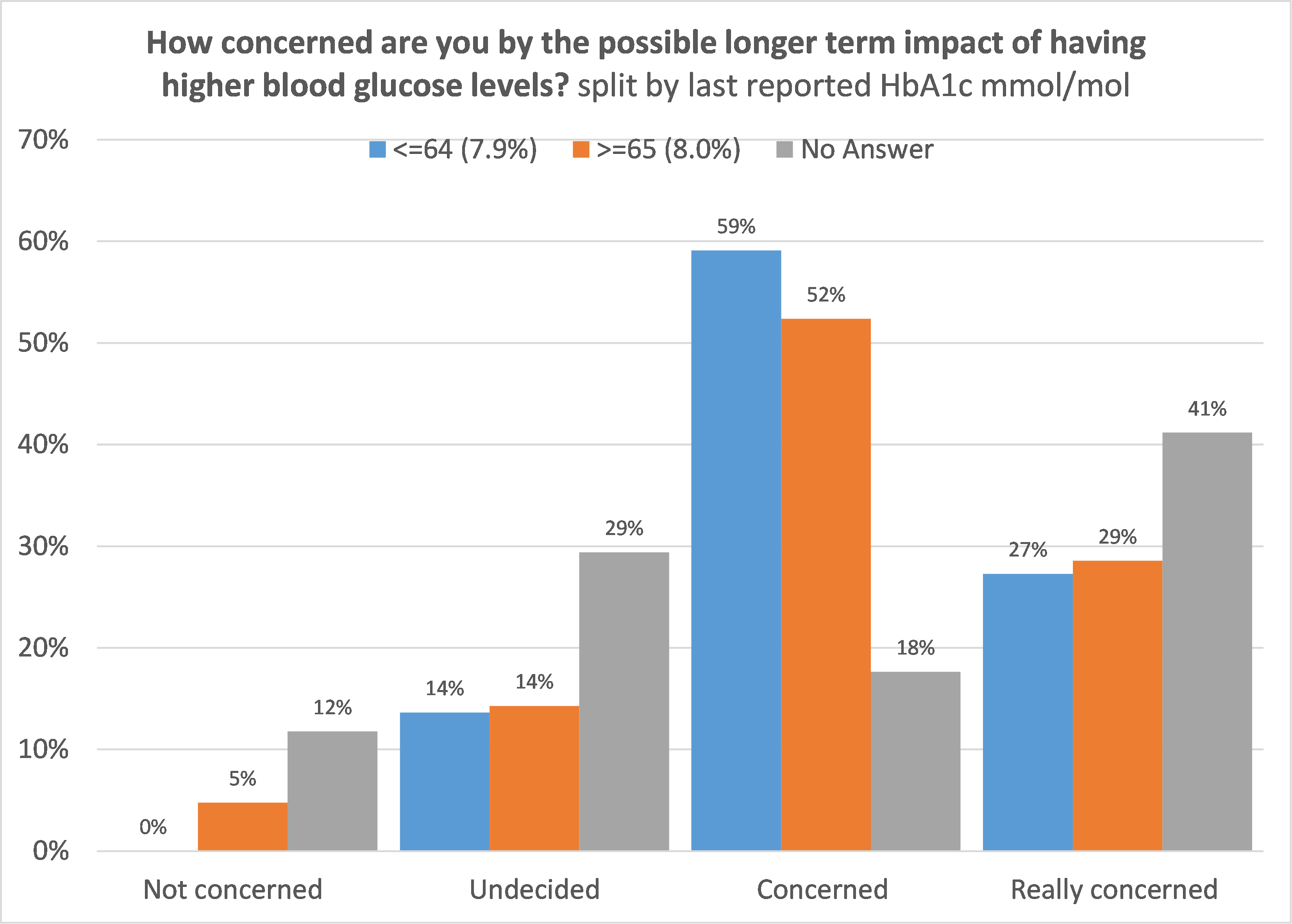

Supplement: sj-tif-2-dst-10.1177_19322968211009261 – Supplemental material for People with Type 2 Diabetes Mellitus (T2DM) Self-Reported Views on Their Own Condition Management Reveal a High Level of Insight into the Challenges Faced [file sj-tif-2-dst-10.1177_19322968211009261.tif]
